# Understanding Trust and Changes in Use After a Year With the NHS COVID-19 Contact Tracing App in the United Kingdom: Longitudinal Mixed Methods Study

**DOI:** 10.2196/40558

**Published:** 2022-10-14

**Authors:** Cecily Pepper, Gisela Reyes-Cruz, Ana Rita Pena, Liz Dowthwaite, Camilla M Babbage, Hanne Wagner, Elena Nichele, Joel E Fischer

**Affiliations:** 1 Horizon Centre for Doctoral Training School of Computer Science University of Nottingham Nottingham United Kingdom; 2 Mixed Reality Lab School of Computer Science University of Nottingham Nottingham United Kingdom; 3 Horizon Digital Economy Research University of Nottingham Nottingham United Kingdom; 4 NIHR MindTech MedTech Co-operative School of Medicine University of Nottingham Nottingham United Kingdom

**Keywords:** COVID-19, tracing app, digital contact tracing, trust, public health, technology adoption, compliance, longitudinal, mixed methods, thematic analysis, mobile phone

## Abstract

**Background:**

Digital contact tracing (DCT) apps have been implemented as a response to the COVID-19 pandemic. Research has focused on understanding acceptance and adoption of these apps, but more work is needed to understand the factors that may contribute to their sustained use. This is key to public health because DCT apps require a high uptake rate to decrease the transmission of the virus within the general population.

**Objective:**

This study aimed to understand changes in the use of the National Health Service Test & Trace (T&T) COVID-19 DCT app and explore how public trust in the app evolved over a 1-year period.

**Methods:**

We conducted a longitudinal mixed methods study consisting of a digital survey in December 2020 followed by another digital survey and interview in November 2021, in which responses from 9 participants were explored in detail. Thematic analysis was used to analyze the interview transcripts. This paper focuses on the thematic analysis to unpack the reasoning behind participants’ answers.

**Results:**

In this paper, 5 themes generated through thematic analysis are discussed: flaws in the T&T app, usefulness and functionality affecting trust in the app, low trust in the UK government, varying degrees of trust in other stakeholders, and public consciousness and compliance dropping over time. Mistrust evolved from participants experiencing sociotechnical flaws in the app and led to concerns about the app’s usefulness. Similarly, mistrust in the government was linked to perceived poor pandemic handling and the creation and procurement of the app. However, more variability in trust in other stakeholders was highlighted depending on perceived competence and intentions. For example, Big Tech companies (ie, Apple and Google), large hospitality venues, and private contractors were seen as more capable, but participants mistrust their intentions, and small hospitality venues, local councils, and the National Health Service (ie, public health system) were seen as well-intentioned but there is mistrust in their ability to handle pandemic matters. Participants reported complying, or not, with T&T and pandemic guidance to different degrees but, overall, observed a drop in compliance over time.

**Conclusions:**

These findings contribute to the wider implications of changes in DCT app use over time for public health. Findings suggest that trust in the wider T&T app ecosystem could be linked to changes in the use of the app; however, further empirical and theoretical work needs to be done to generalize the results because of the small, homogeneous sample. Initial novelty effects occurred with the app, which lessened over time as public concern and media representation of the pandemic decreased and normalization occurred. Trust in the sociotechnical capabilities of the app, stakeholders involved, and salience maintenance of the T&T app in conjunction with other measures are needed for sustained use.

## Introduction

### Digital Contact Tracing and the COVID-19 Pandemic

Throughout the COVID-19 pandemic, mobile apps enabling digital contact tracing (DCT) became a widespread solution adopted by many countries worldwide for mitigating the spread of SARS-CoV-2 [1,2]. A need for understanding how people feel toward these apps arose once they started being announced and released from 2020 onward. Several studies investigated people’s perceptions and attitudes in relation to the acceptance and adoption of DCT [3-6], privacy and ethical concerns [7-9], and the type of messaging used to promote the apps (eg, targeting individualism or collectivism) [7,10]. Although conducted using hypothetical apps and scenarios, early studies found that large percentages of the people consulted said they would use the apps if they became available and that, initially, many people had positive perceptions toward DCT as a medium for minimizing the spread of the virus in society [3,11].

However, after 2 years of the pandemic, the overall uptake of DCT apps remained low, especially because they were mostly voluntary [2]. This imposes a technical challenge given that some studies suggest that the apps need a high uptake rate (from 56% to 95%) to work effectively [12,13]. However, other theoretical and empirical studies found that effects can also be seen at lower adoption levels. For instance, research has estimated that >32% uptake can be helpful in lowering the epidemic to manageable levels when the infection rate is moderate [14]. Other work has estimated that cases could be reduced between 0.8% and 2.3% for every incremental percentage point of app uptake [15]. Nonetheless, regardless of infection rates, higher uptake would represent a higher number of cases averted [15], which makes the case for better understanding DCT app uptake and further incentivizing them.

An intention-behavior gap has been identified in DCT uptake [11], but little is known about the *actual* experiences of people with the apps [16], especially over time, as there have been only few longitudinal studies in this regard [8]. Privacy has been found to be a major concern of people, possibly hindering app uptake [6,17], but the “privacy paradox” has been also acknowledged and investigated, where although people state being highly worried about their privacy, they do not act on their concerns [18]. Thus, more work is needed to understand people’s experiences with DCT and other factors, beyond privacy concerns, that may be involved in their adoption and sustained use over time. In the context of DCT, sustained use can be understood in terms of passively engaging with the specific characteristics of the system such as having Bluetooth tracing enabled and taking part in more active aspects of contact tracing such as checking in to public places.

This study builds on previous explorations of trust in DCT apps [19], where trust has been found to be a major factor in their intended adoption, examining in further detail people’s experiences and trust perceptions—in relation to a DCT app and its related ecosystem—and their relationship to DCT adoption and use over time. In this study, ecosystem is defined as the set of stakeholders involved in developing, maintaining, and using the Test & Trace (T&T) app. Trust is a complex, context-dependent subject [20], being considered as a mental state felt by people toward others (people, systems, organizations, etc) [21,22]. Regarding technology, trust in stakeholders who control it is often a prerequisite for trust in the technology itself [22]. In the context of DCT, this effect was also shown in research by von Wyl et al [23], where trust in the Swiss government and health authorities was positively correlated with app uptake. Trust therefore plays an important role in technology acceptance uptake.

### Technology Acceptance Concepts

There exists a series of models of factors influencing acceptance, with the technology acceptance model (TAM) [24] being one of the most well-known models, which includes factors such as perceived usefulness and perceived ease of use. Specific models for health care informatics applications have also been developed such as the health information TAM, which includes factors such as health status, health beliefs and concerns, and perceived health threat [25]. In this study, we draw from the technology acceptance lifecycle (TAL) by Nadal et al [26], which creates terminologies to take into consideration the temporality of technology acceptance by dividing it into three main stages: before use acceptability (before the first use), initial use acceptance (after the first use but before adoption), and finally, sustained use acceptance (after adoption). Initial use is related to the human-computer interaction literature concept of the novelty effect (NE), which is the set of responses to initially using technology but that does not equate to the long-term use pattern [27], or sustained use under TAL terminology.

Several studies on NE have shown that as it “wears off,” many users stop using the technology [28,29]. In an activity tracker long-term use study [29], research showed that only curiosity about the technology and data is not enough to provoke sustained use, which tends to be associated with personal and social motivation as well as gaming motivation. However, studies of the NE in health informatics are related to the user’s own health as opposed to individual action for public or collective health, which is the case for DCT in the context of the COVID-19 pandemic, which will be explored in this paper.

### Context of the Study

In this study, we focused on the National Health Service (NHS) COVID-19 T&T app, which is the national DCT app for England and Wales. The T&T app was launched in England and Wales on September 24, 2020. The app uses Bluetooth for contact tracing by recording locally on a smartphone the amount of time spent with, and distance between, users. If a user has been in close contact with someone who tests positive, the app will notify the user and give guidance. Furthermore, the T&T app also allows one to check in symptoms, book tests, and input test results as well as checking in to various places such as hospitality venues (eg, pubs or restaurants) by scanning the venue QR code onto the app. In this study, we consider sustained use as engaging with the above features, both in a passive (eg, having Bluetooth tracing on) or more active (eg, checking in at venues) manner. The T&T app had a total of 20.35 million downloads (data from December 2, 2020) at the approximate time of the first survey and 28.76 million (data from November 3, 2021) at the time of the second survey and interview [30].

**Figure 1:**
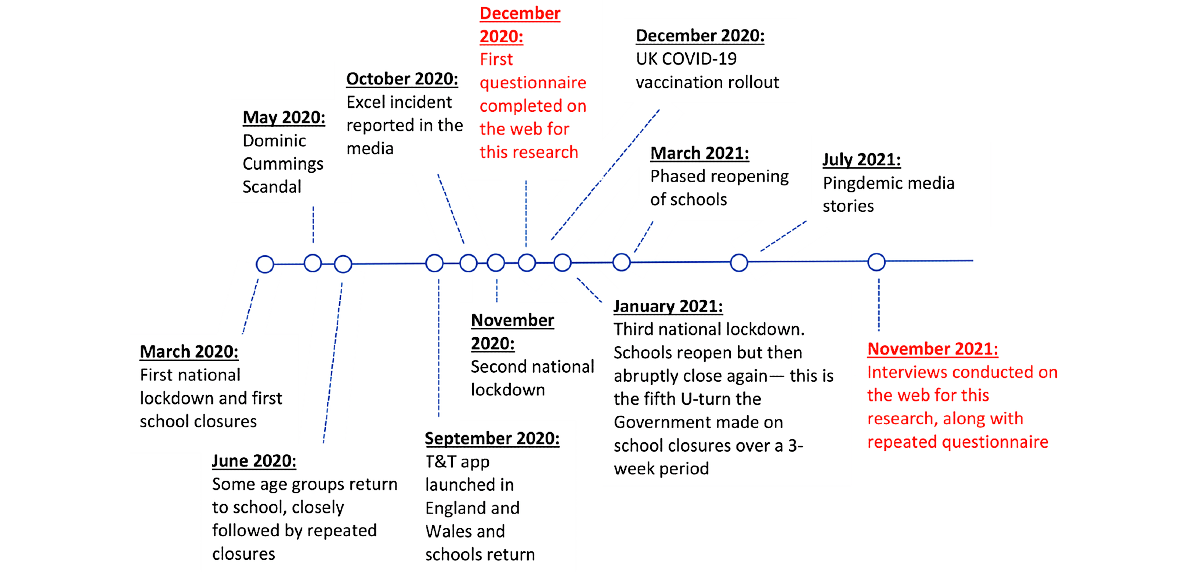
A timeline detailing the key events that are referred to in this research. T&T: Test & Trace; UK: United Kingdom.

As the T&T app was designed for use within the United Kingdom, a breakdown of COVID-19–related events, lockdowns, and measures is necessary for context. The first lockdown in the United Kingdom occurred in March 2020, with lockdown measures being legally enforced soon after [31]. In September 2020, the T&T app was launched in England and Wales with the hope to “help control coronavirus (Covid-19) transmission” [32]. Multiple lockdowns followed in November 2020 and January 2021 [31]. Throughout the pandemic, multiple school closures and reopenings occurred, with the UK government making several U-turns in their decisions [33]. The COVID-19 vaccination rollout began in December 2020, starting with the most vulnerable and then gradually extending through age groups and risk levels [34] (see the timeline in Figure 1).

Along with providing context for the lockdowns and measures of the United Kingdom, several media events were also significant in the public’s perception of the T&T app. In May 2020, a scandal occurred with the prime minister’s chief advisor, Dominic Cummings, who faced public outrage and calls to resign after driving across the country during a UK lockdown [35]. Several months later, in October 2020, shortly after the launch of the T&T app, the media reported an error by Public Health England, who had been using Microsoft Excel to store public health data and an error in formatting resulted in >15,000 unreported cases of COVID-19 [36]. In addition, as the public continued to use the app to check in to venues, a surge in “pings” (notifications that tell the user they need to self-isolate) occurred in July 2021 that was labeled by the media as the “pingdemic.” This caused multiple issues with manufacturing and hospitality, especially because there was a legal duty to self-isolate if the user was pinged [37]. The timeline (Figure 1, inspired by the timeline from the Institute for Government [31]) displays the key events as discussed: the 3 national lockdowns, school closures, and reopenings; 3 significant media scandals; and the 2 data collection points for this research.

This longitudinal study builds on the research by Dowthwaite et al [19], which was a quantitative study that investigated attitudes and trust toward the T&T app. To understand this further, this study aims to explore how such attitudes and trust changed or were maintained over time by analyzing research data collected approximately a year apart.

## Methods

### Research Design

This research had a longitudinal design as questionnaire data were collected at 2 time points roughly a year apart, with answers being explored in detail in an interview at the second time point. Therefore, the research had a mixed methods design, gathering quantitative data from the questionnaires and qualitative data from the interviews. The analysis makes use of the quantitative data as a backdrop but emphasizes the qualitative data collected to fulfill the aim of exploring the reasons for any changes that may have occurred over time in attitudes, use, and trust toward the T&T app.

### Ethics Approval

Ethics approval was granted by the research ethics committee of the authors’ institutions (approval number: CS-2020-R80 for the interviews and CS-2020-R10 for the questionnaires).

### Participants

To study changes in attitudes toward the T&T app, participants who took part in a questionnaire survey in December 2020 were invited to participate in an interview approximately 1 year later to see if and how their question responses may have changed and why. Participants were recruited via email and social media through the authors’ personal and professional networks (eg, listserv mailing lists, Twitter, and Facebook) to participate in a web-based questionnaire survey and were asked to consent to being contacted for a follow-up interview. A total of 48 people began the questionnaire, with 40 complete responses. From these respondents, 16 agreed to be contacted. Interviews were conducted with 9 participants who responded when contacted in November 2021. They were aged between 29 and 49 (average age 38, SD 8) years. Of 9 participants, 3 (33%) were men, 4 (44%) were women, and 2 (22%) were nonbinary (Table 1). We did not explicitly ask about occupation, although some participants discussed it in their responses.

**Table 1:** Basic demographics of participants (N=9).

Demographic		Participants, n (%)
**Gender**		
	Man	3 (33)
	Woman	4 (44)
	Nonbinary	2 (22)
**Highest educational level**		
	Undergraduate degree	1 (11)
	Master’s degree	7 (78)
	Doctorate	1 (11)
**Employment status**		
	Employed full-time	4 (44)
	Employed part-time	2 (22)
	Student	3 (33)
**Ethnicity**		
	White	8 (89)
	Asian	1 (11)
**Religion**		
	None	5 (56)
	Christian	2 (22)
	Muslim	1 (11)
	Other (not specified)	1 (11)

### Materials and Procedure

Participants answered the same questionnaire described in an earlier study in this journal [19], at 2 time points, between November 13 and December 23, 2020, when the United Kingdom was between “lockdown 2” and “lockdown 3” and subject to a regional tier system, and between October 25 and November 5, 2021, when most restrictions across the United Kingdom had been lifted. The first questionnaire was administered on the web, whereas the second was administered as part of an interview. At the start of the web-based questionnaire (Multimedia Appendix 1), participants were provided with information and privacy notices and gave informed consent to participate. Questions took the form of either multiple choice or Likert and Likert-like scales, except for a single open-ended question that was included to elicit further comments. The first part of the survey asked participants to indicate what knowledge and experiences they had of COVID-19 and the NHS T&T app; for example, compliance with any requests to self-isolate, whether they had downloaded the app, and if not downloaded, then why not. Those who had downloaded the app were then asked for their reasons for downloading and experiences of using the app. They were then asked about the technology and functionality of the app, including perceived usefulness and ease of use, understanding of how it worked, and the importance of features such as opting in and out of contact tracing. Finally, they were asked about the levels of trust in distinct aspects of the app, including responsibility, security, reliability, functionality, data use, and stakeholders and wider society.

**Figure 2:**
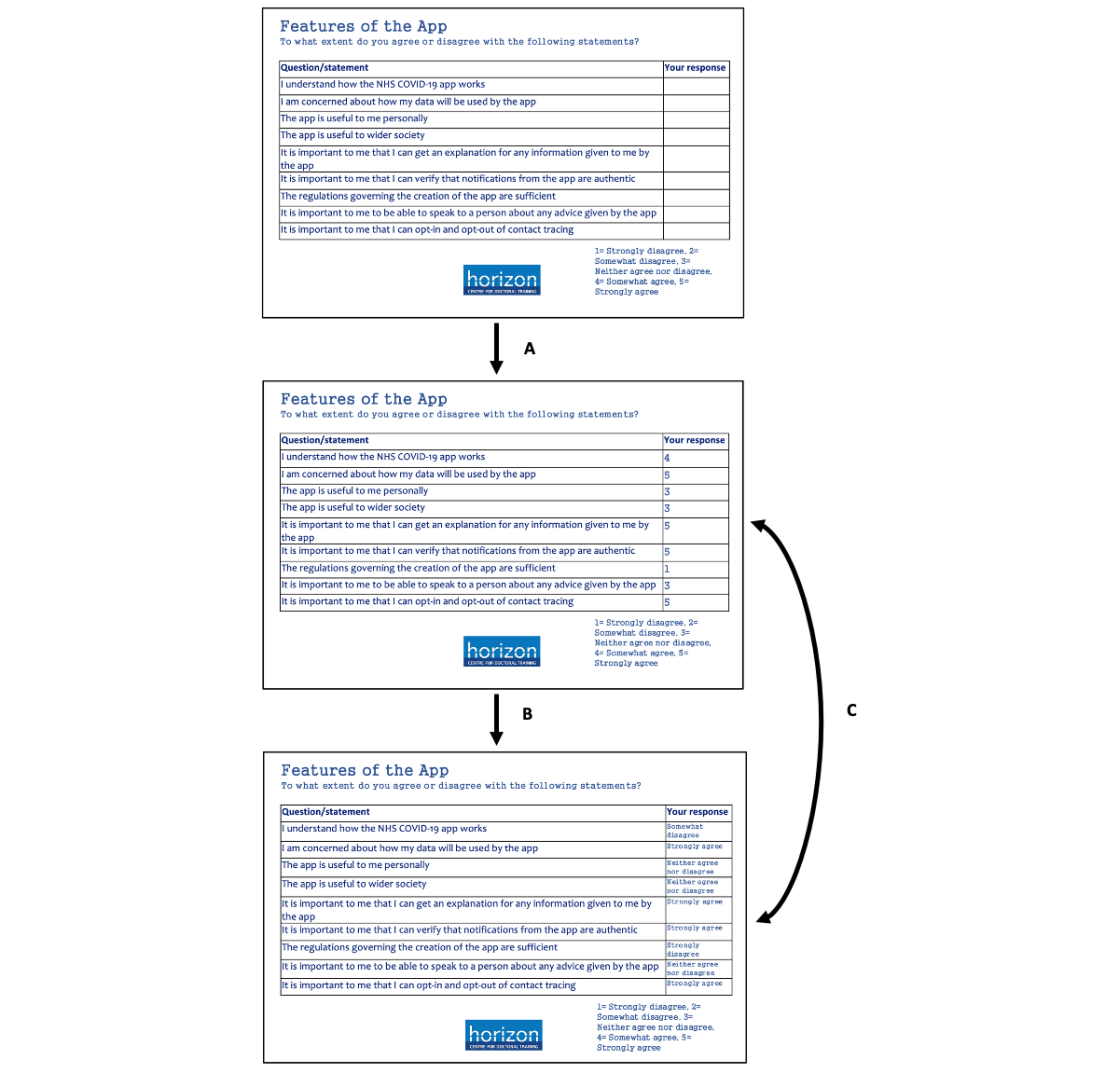
Example of a question block displayed to participants. After being shown the blank slide, they fill in their current answers in November 2021 (A). After this, they are shown their previous responses from December 2020 (B) and they can refer back between slides as visual reminders of their responses during their interviews as indicated by (C). NHS: National Health Service.

The web-based interview, approximately 11 months later, lasted between 31 and 56 (average time 44) minutes. After confirming their consent to the interview, the interviewer started recording the session and shared their screen with the participants. The questions from the earlier questionnaire were shown to the participants, presented in groups as they were in the original survey, without their previously provided responses (Figure 2). Participants were asked to respond to the questions as they would now, and these responses were added to the slide; then, they were shown their original responses of the year before. Any changes were then probed; for example, why they felt more positive or negative or why they thought they responded a particular way originally but not now. This was done to provide a visual reminder to the participants of the questions and their responses, which could easily be compared between slides, and to provide a focus during the discussion. Following this, they were asked to summarize how their experiences with the T&T system had changed since they filled out the questionnaire, how they feel about the app and T&T, and how their actions had changed over the previous year. They were also asked how their trust in T&T had changed and whether the rollout of vaccines affected this. Finally, they were asked to highlight any specific media stories, events, or other factors that had affected their trust in T&T.

### Analysis

Interviews were recorded through Microsoft Teams and transcribed through a 2-step process: first, by generating initial automated transcriptions through Microsoft Stream and then by thoroughly listening to the interview recordings and revising the transcripts to ensure accuracy.

**Figure 3:**
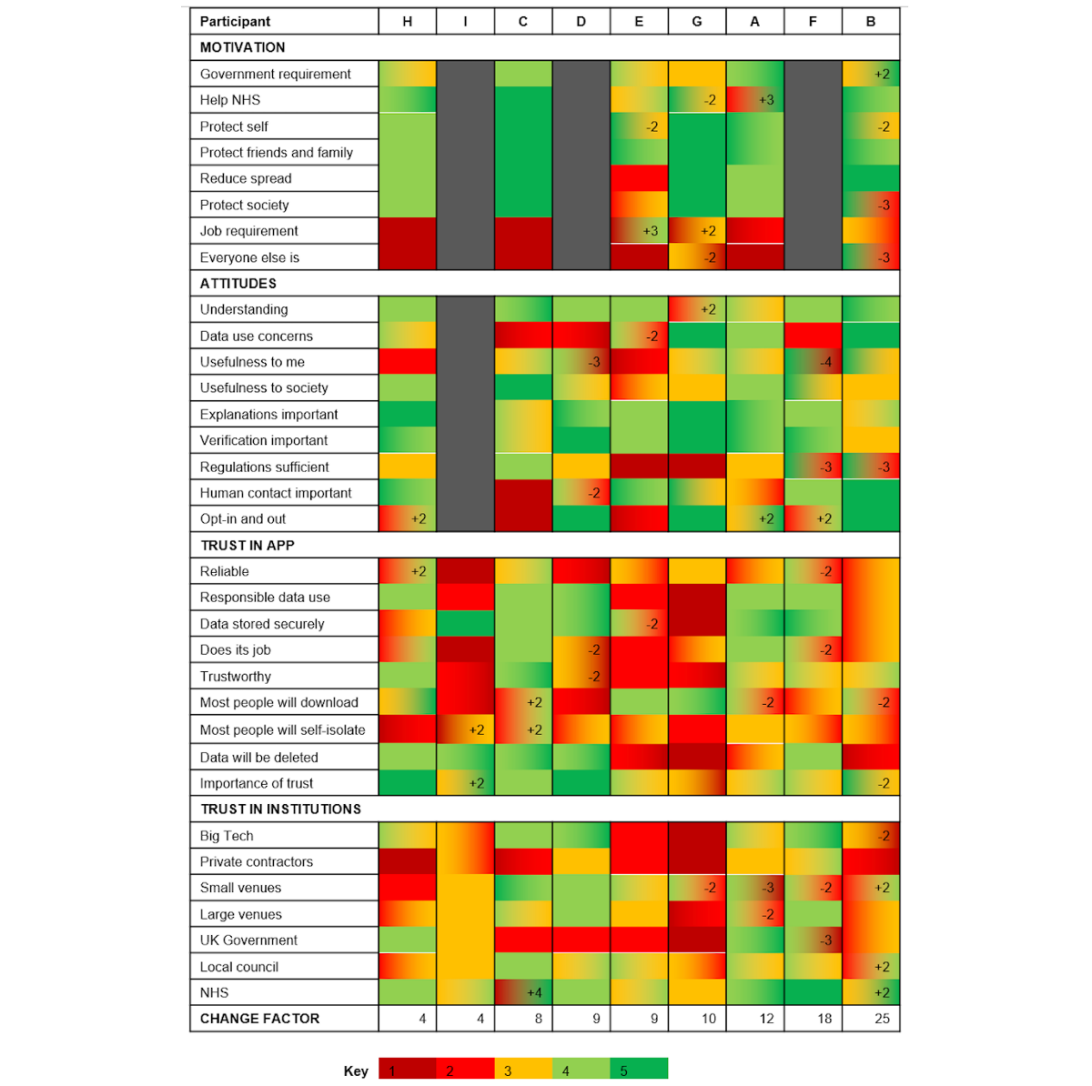
Change in questionnaire responses between December 2020 and November 2021 by the participants. Dark red (1) for strongly disagree up to dark green (5) for strongly agree and gray for not applicable (for participants who did not have the app installed at the point of interview). Positive and negative changes of ≥2 points are indicated by integer and color gradient. Participant columns are ordered by ascending change factor, which indicates the sum of ≥2 change points by the participants. NHS: National Health Service; UK: United Kingdom.

The initial questionnaire data from December 2020 were downloaded to a spreadsheet, and after the interviews, new responses to each question were added. The difference between initial and second responses was calculated numerically for statements where possible (Figure 3). No other summary statistics or any inferential statistics was calculated because we were interested in the within-participant changes in response. Because of the nature of repeated responses, where minor fluctuations in response may be expected day-to-day, it may reasonably be expected that a change of a single point (eg, strongly agree to agree) may not represent an actual change in opinion; only changes of ≥2 points are reported. This means that a participant would have at least changed from strongly agree or disagree to neutral, agree to disagree, or vice versa. A change factor was reported (Figure 3) by calculating the sum of each participant’s change points, regardless of the direction, to summarize the amount of general change in trust and perceptions of the T&T app each participant had experienced over time.

The first 3 authors conducted a thematic analysis of the interview data by following the 6 phases established by Braun and Clarke [38]. The data were analyzed from an experiential perspective with the aim to capture and explore people’s own perspectives and understanding [39]. The 3 authors familiarized themselves with the data by (1) first correcting 3 interview transcriptions each and then reviewing the whole data set and then (2) inductively generated initial codes aiming to capture both semantic and latent meanings. The authors used a list of common terms characterizing the interviews for creating the codes, but no predefined concepts were searched for while coding the data. The process was conducted using Microsoft Excel through an index system to track each code generated and match it with the corresponding raw data. The authors then (3) reviewed each other’s work once all interview transcriptions were coded and collaboratively searched for candidate themes by collating the codes, which then (4) were reviewed and developed into themes and subthemes. These were (5) named and defined and (6) elaborated at length in writing, selecting relevant data extracts for illustrating them.

The 3 authors kept a reflexive journal throughout and kept ongoing reflective discussions for considering possible biases and personal perspectives in relation to T&T and the overall pandemic experience to be aware of their influence on the analytical process. All 3 authors had different personal experiences of using the T&T app, from never downloading the app, to experiencing flaws, and continued use; hence, the overall perspective on the T&T app felt balanced. The collaborative analysis further enabled a more nuanced understanding of the data, rather than aiming for a consensus about the meaning [38].

## Results

### Summary of Responses and Themes

The comparison of responses given by the participants on the 2 study time points (December 2020 and November 2021) is summarized as a chart indicating positive, negative, and no changes, as well as the overall change factor (Figure 3). Participants D and F deleted the app between the initial questionnaire study and the interview study; participant I never downloaded the app and did not intend to do so; the remaining participants downloaded the app and still had it on their phones at the time of the interview. Further details about the participants, such as demographics and a summary of individual responses and changes, are provided in Figure 2 and Multimedia Appendix 2.

A total of 6 themes were developed (Multimedia Appendix 3) from the analysis of the 9 interviews. As the interview transcripts were coded inductively, the sixth theme was not entirely relevant to the research aims; therefore, in this study, we concentrated on elaborating 5 themes relevant to the research objectives (as stated in the *Introduction* section). Themes revolved around the T&T app as experienced by participants, who highlighted several *encountered and perceived flaws* (T1) and reported *different degrees of trust* in it (T2). Themes further developed around participants’ trust in stakeholders, with reported general low *trust in the government* owing to their poor pandemic handling (T3) and a mix of *trust in other stakeholders* based on their perceived competence and intentions (T4). The final theme revolved around participants’ *changing experiences over time* in relation to the T&T app, their general compliance and trust in stakeholders, as well as decreased exposure and reports observed over time (T5). In the following sections, the 5 themes are elaborated on, including participants’ quotes and references to their changed or maintained responses (Figure 3).

### T1: Flaws of the T&T App Perceived and Experienced by Participants

#### 1a: Lack of Technical Advance and Lack of General Public Uptake

Participants mentioned practical issues related to the proximity measures of the app relying on Bluetooth, including not having it on all the time and its effectiveness in helping track exposure to the virus, especially in complex social contexts. Trust over time that the app is reliable and does its job is broadly negative to neutral (Figure 3):

I’m not sure that the proximity of devices like smart phones is a good indicator of risk of exposure to an airborne virus.

Participant E; motivation of use for self-protecting has decreased; change factor of 9

And there were so many cases of people I knew being told to self-isolate who had absolutely no contact…Yeah, you could be next door neighbours with somebody and have no contact with them and still get told to self-isolate and you had no come back on that.

Participant F; the app’s perceived usefulness to self has decreased by 4 points and trust in app reliability and effectiveness decreased by 2 points; has deleted the app; change factor of 18

Without contextual information, people can’t make good choices about what the information means. So, the stories that came out very quickly about phones being left outside of lockers in hospitals, and NHS staff being pinged because they didn’t turn the app off...You know, people who were getting pinged from across closed walls and things.

Participant I; initial negative perceptions of the T&T app that were maintained over time; never downloaded it; change factor of 4

Moreover, in the view of the participants, the existence of these issues has contributed to the general public abandoning the app. Trust over time that most people will download and self-isolate is broadly negative to neutral (Figure 3):

If people are not using it, then the T&T app it’s like useless.

Participant A; general positive motivations and attitudes toward the app but decreased belief that people will download it; change factor of 12

It’s just technology that is so novel and that it breaks very easily and also people can just choose to not use it, so there’s many obstacles for it to be efficient.

Participant B; decreased motivation of use to protect self and society and decreased belief that others influence own actions and others will download the app; change factor of 25

#### 1b: Lack of Consideration of User Diversity

The participants expressed that the design of the app did not consider different sets of users and their specific needs and situations. For example, there were sociotechnical flaws arising from the unique conditions of frontline workers, as mentioned by participant F:

Because I was every day in school with kids, they weren’t testing. We were basically hung out to dry…I think our situation was so different. If I had it [the phone] on all the time in school, I’d have been pinged so many times to self-isolate.

Participant F; decreased perceived usefulness to self by 4 points and decreased trust that app is reliable and effective by 2 points; has deleted the app; change factor of 18

Participants also mentioned other sets of users who were not considered by the app designers, such as people sharing workspaces and households and varying levels of smartphone users. Participant I summarized this as follows:

And to me the test and trace app was designed with such a Tory model of the world [i.e. this could be interpreted as a conservative and upper-class view], that smartphone users were professionals, that they weren’t frontline users, and that they wanted information or would use an app. That the idea that it would be older users with older phones, that it would be people without the latest smartphone, that it would be people who would have a difficulty navigating smart phones. I just felt like there was so many missed opportunities to have a model of a user who didn’t fit, who Downing Street [UK government] was clearly designing this thing for.

Participant I; initial negative perceptions of the T&T app that were maintained over time; never downloaded it; change factor of 4

#### 1c: T&T App Designed for Individuals Rather Than a Collective Effort

For participants, adherence to the guidance and general uptake of the T&T app as a collective action was needed for effectiveness. However, some participants considered that it was designed for individual action and responsibility instead of creating an app modeled by mutual aid:

Having the app puts the responsibility of using it on the individual, it’s like it’s not a communal or collective solution. It’s very much like, does this person have a phone that can support this? Does it have Bluetooth well enough? Does the location tracking work? Is there enough battery for people who have an older phone? Or maybe do not even have a phone at all.

Participant G; initial mistrust of the T&T app that remained unchanged over time; change factor of 10

Participants felt that the T&T app did not help society in general, but simply their closest circle, and that it made people lose their goodwill in the general public:

I don’t really know what that means completely, I feel like I’m helping only my small circle as much as I can, but I don’t think I can affect like the broader society.

Participant B; decreased motivation of use to protect self and society; change factor of 25

#### 1d: Lack of Clarity and Certainty in Understanding of the T&T App

When prompted to talk about how the T&T app works, there was a lack of certainty in the participants’ answers. In the survey, they reported a good understanding of the app and strongly agreed with the need to have explanations and verifications by the T&T app (Figure 3). While some participants mentioned that decisions were made by a combination of the T&T app and humans, other participants mentioned that the decisions were made only by the T&T app. A few participants were in both groups as their answers depended on the specific context discussed (eg, the existence of regional clusters will be handled differently). The participants mentioned their lack of understanding and certainty regarding how the app works and the extent of human involvement in the system:

I’m also realising how embarrassingly little I know about how this app works...but my assumption is like almost all automated. I just feel like somewhere in it someone’s got the ability to be like let’s send a message.

Participant H; mix of positive and negative responses that remained broadly the same; change factor of 4

You know what? I don't know. I think originally, it was humans and the app, but I think they may have made it more—I'm going to say app only…There are certainly people in the system. But come to think of it now, I think I'm actually very sure they're probably not involved in decisions as to whether or not self-isolate someone.

Participant C; reported a good understanding of how the app works that was maintained over time; change factor of 8

#### 1e: Suggestions Given to Improve the T&T App

As the participants discussed which aspects of the T&T app were seen as flawed, they also gave suggestions on what could be improved. Most of the suggestions were related to messaging and communication:

I feel like it could have been designed around…positive feeling. Here’s a little something “Oh it’s cool that you’ve checked in” or “You checked in five times.” Not to gamify everything, that’s also really bad, but I think just a little “Thank you for having on contact tracing” that “You’ve been outside, you are good, remind your family and friends to do that too”...that would have been nice.

Participant G; maintained motivations and attitudes to protect self and others; change factor of 10

There were also calls for the T&T app to be more human-centered with simpler and manual check-ins and to help the human T&T team with options to verify the app to avoid scams.

### T2: Trust in the T&T App Differs Based on Perceived Usefulness and Functionality

#### 2a: Varying Degrees of Trust

Trust of the participants was primarily based on app functionality and effectiveness. Thus, if the participant believed these factors to be flawed, then trust was low, whereas if the app was thought to be effective, then trust was higher. For example, some participants saw media stories such as the “Pingdemic” (Figure 1) and the fact that real consequences occurred such as shutting down venues as signs that the app was working effectively and thus trust was higher. Whereas other participants thought that the app had poor functionality and effectiveness, which led to a loss of confidence and trust:

The “Pingdemic” in my mind just shows the app was doing its job because you know Covid was on the rise and people were getting pinged for it. So, I suppose yes, in that case, I probably do think that it’s doing its job.

Participant C; initial trust in the app that was maintained over time; change factor of 8

Whereas I think if it had worked properly and people had trusted it, then maybe we’d be in a better situation now.

Participant F; decreased perceived usefulness to self by 4 points and decreased trust that app is reliable and effective by 2 points; has deleted the app; change factor of 18

Your trust is very surrounded by the fact that it works or not.

Participant I; initial negative perceptions of the T&T app that were maintained over time, never downloaded it; change factor of 4

#### 2b: Flawed Development and Lack of Explainability and Transparency

This subtheme links to the TAM [24] constructs: perceived usefulness and perceived ease of use. As the T&T app was seen as flawed in both aspects by participants, their acceptance and trust in the app were low. Reasons given for mistrust in the app included flaws such as the T&T app creators’ lack of understanding and consideration of all the complex factors needed for the app to be effective and the lack of explanations behind notifications:

You know if there could have been any explanation as to why I was being pinged and my husband wasn’t. That would have really helped.

Participant D; decreased perceived usefulness to self and decreased trust in app effectiveness and trustworthiness; has deleted it; change factor of 9

I mean, it doesn’t bother me that an outside agency built the app. I just don’t think they built it very well...I don’t think they knew what they were doing...in their ability to understand the socio-technical complexities of the assemblage that needs to be put in place for this thing to work.

Participant I; initial negative perceptions of the T&T app that were maintained over time; never downloaded it; change factor of 4

#### 2c: Human T&T, Physical Measures, and Human Guidance

Participants had a strong view that human T&T and physical measures, such as mask wearing, social distancing, and ventilation, were more trustworthy than the T&T app because the app was fundamentally flawed. In addition to higher trust, there was also a sense of control gained from complying with physical measures, which in turn made participants feel safer:

It might be a side effect of this pandemic, that we’re all looking perhaps for a little bit more control. But also, it’s going to give me a result which I have more confidence in, particularly doing regular testing...Whatever is the result of my weekly tests, that’s something which I know I can act upon or deal with, whereas with a ping, all that is, is basically a cause of stress really.

Participant D; decreased perceived usefulness to self and decreased trust in app effectiveness and trustworthiness; has deleted it; change factor of 9

Human contact in the T&T app was regarded as important (Figure 3). This was done for multiple reasons, such as ensuring accountability, verifying automated decisions, and avoiding unnecessary actions such as self-isolating:

There’s an element of it where I think anything that’s algorithmic or AI or machine learning...I want some human accountability somewhere in the chain. And that’s more of a principle thing. It’s kind of less about the app, it’s more about like I’m fine with you using AI to improve the service but I still need some accountability and some ability to say, “well, why was this decision reached? How was this decision reached?” And its impact on me. I think a human [needs to be] somewhere in that system.

Participant H; mix of positive and negative responses that broadly remained the same over time; change factor of 4

### T3: General Low Trust in the Government Owing to Poor Pandemic Handling, Including Procurement of the T&T App

#### 3a: Inconsistent Decision-making and Lack of Compliance

Participants felt anger and frustration toward the UK government because of mixed messaging and the perceived hypocrisy of making rules yet not following them. This, along with perceived poor and inconsistent decision-making throughout the pandemic, led to participants’ low trust in the government:

I think all the mixed messaging, basically saying “now Covid's your problem, you decide if you want to wear a mask or not,” I just think all of that plus an app which is unreliable just makes you not feel particularly trustworthy.

Participant D; initial negative trust in the UK government that has remained unchanged and decreased trust in the app; change factor of 9

I think over the last year we’ve had a number of cases of political leaders in England and the UK...who appear to have either not been following the rules or have been following them loosely, shall we say, and I think that would have affected trust around these things.

Participant E; initial negative trust in the UK government that has remained unchanged; change factor of 9

#### 3b: T&T App Creation and Data Management

Another reason for reported mistrust in the UK government was the doubt around the creation of the app and how the data were collected, stored, and processed. Several aspects reported in the media, such as the Excel incident, technical decisions, and money spent, in addition to the UK government’s history of failures with technology projects, all resulted in a general mistrust in the government and questioning of their intentions regarding data management:

Because I am a data scientist, so I know what kind of data people use. I just wouldn’t want this to be an exercise of the government to collect free data under the excuse that it’s for the national security. Yes, I just don’t really know who and where this data will be stored considering the problem that happened to that Excel file. It just throws a shadow over anything else.

Participant B; decreased trust in Big Tech; change factor of 25

The government and big IT projects, I mean, you know there’s been a history of failure there, hasn’t there? But I just think this was so important to try and get it right and they didn’t.

Participant D; initial negative trust in the UK government that has remained unchanged and decreased trust in the app; change factor of 9

#### 3c: Tensions Between Enforcing the T&T App and Opting Out of Tracing

Within this subtheme, participants expressed morally complex views about their ability to opt in and out of contact tracing. Although participants believed that the freedom to opt-out of contact tracing was important, they also believed that this was a complex decision as the T&T app requires contact tracing to be turned on to be effective. Furthermore, the importance of not only user control within the T&T app (being able to opt-out) but also the need for transparency was highlighted:

It’s good for people to have a choice. I mean, yeah, just for people to have a choice to have this contact tracing or not where I ideally—so I think it should be mandatory—so that the virus is not spreading. But at the same time people should have the choice to opt in or out I think.

Participant A; increased belief that opt-out should be an option; change factor 12

For me the whole point of having the app is for the contact tracing. If I was turning off contact tracing, I might as well uninstall the app.

Participant C; initial disagreement with the idea of opting out of tracing was maintained over time; change factor of 8

#### 3d: General Disapproval of the Government's Actions During the Pandemic

This subtheme was substantial and included a variety of reasons why participants disapproved of the government’s actions over the course of the pandemic. A common opinion among the participants was that the government handled the pandemic poorly and could have implemented the T&T app more effectively. The measures suggested by the government were also thought to be elitist in principle and punished the diverse UK population, including the working class and frontline workers. In addition, the government was considered overconfident, which led to a premature relaxation of rules, with multiple participants reporting that rules and enforcement of rules should have been stricter to improve compliance and reduce case numbers. This was accentuated by some participants’ comparison of the England T&T system to pandemic handling in Scotland, which was thought to be clearer and stricter and thus more effective:

I was thinking I didn’t agree with how the UK Government handled Covid in general...This is a lay person’s opinion, but I would have liked much more conservative approach, let’s say closing down earlier. Much more money for people to stay home. Extended furlough. Everybody gets Universal Credit. That’s what I would have done but I didn’t have to crunch the Excel sheet, so it’s easy for me to say that.

Participant G; initial low trust in the government that remained unchanged; change factor of 10

### T4: Varying Degrees of Trust in Stakeholders

#### 4a: Relationship Between Perceived Intentions and Competence

Participants’ reported trust in the rest of the stakeholders (ie, private contractors, Big Tech, large and small hospitality venues, local councils, and the NHS) varied in a spectrum of their perceived intentions and competence exhibited throughout the pandemic:

If we’ve got a spectrum of competence and then good intentions, Serco, Capita et al. private contractors, they’re ranking dead last on both criteria. I regard them as both incompetent and malicious. Tech companies I regard as competent and malicious. So, they end up in the middle and then with small hospitality venues, not malicious, like none of the rest really I’d put down as particularly malicious. I’d say that chain restaurants are largely malicious...So for them, I’m measuring on a competence basis...For NHS they get a four because of some worries on competence, the UK Government get a four because of some worries on maliciousness. There’s, there’s a chart here.

Participant H; mix of trust in stakeholders; change factor of 4

**Figure 4:**
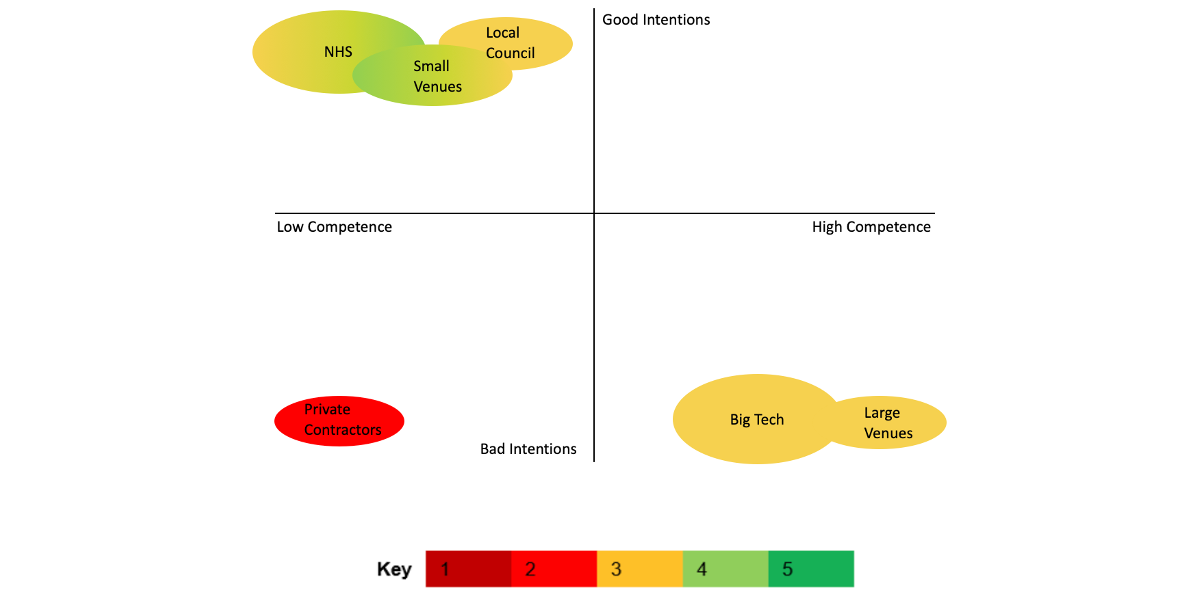
Overall reported trust in stakeholders based on their perceived intentions and competence. Figure created based on data from Figure 3. Color key represents average trust across participants (where decimals were rounded to the closest integer) at the 2 time points. Gradient represents change of average trust over time (from left to right), and solid color represents no change in average trust over time across all participants. The size of colored areas aims to illustrate the number of participants that mentioned the stakeholder in question. This figure is meant to illustrate generalized perceptions gathered from interview and survey data and does not represent statistical measurements found in the data. NHS: National Health Service.

**Figure 5:**
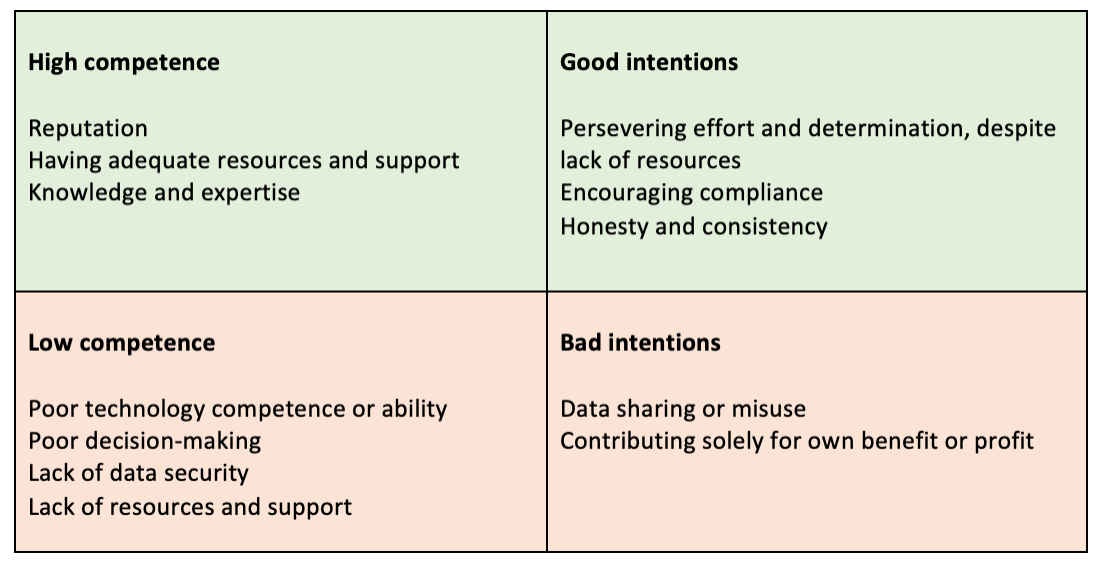
Participants’ positive and negative views of stakeholders that influence participants’ trustworthiness.

Building on this categorization stated by participant H, further subthemes were developed in which participants reported mixed feelings regarding trust in stakeholders. These feelings of trust or mistrust depended on stakeholders’ perceived intentions and competence, as expressed by the participants (Figures 4 and 5). It is worth noting that these figures represent the generalized perceptions of participants’ views regarding stakeholders and are not intended to be an objective grading or make any assumptions about stakeholders’ actual competence or intentions. In addition, these are not equally weighted reflections of the views of all participants involved in the research, as not all participants mentioned all stakeholders, but rather are used here to illustrate participants’ general reasoning for trusting—or not trusting—such stakeholders.

#### 4b: Big Tech, Private Contractors, and Large Hospitality Venues

Overall, participants reported mixed feelings regarding Big Tech and large hospitality venues, as their capabilities for managing T&T were regarded as stronger in comparison to the rest of the stakeholders. Some participants mentioned their reputation, expertise, infrastructure, and level of involvement in T&T, all of which provided a general sense of high competence despite the known mistakes and issues arising throughout the pandemic:

The bigger you are, if you’re a larger venue you might have like a, you know, data control officer...you know, you’re large enough to have a corporate policy on it, you know there’s at least 50/50 chance that was followed in your particular franchise, so I just think the odds of it being handled appropriately increase once you’re a large chain just cause you’ll have that corporate infrastructure underpinning it.

Participant H; neutral to negative trust in large venues; change factor of 4

Nevertheless, participants expressed general concerns about those main stakeholders (Big Tech, private contractors, and large hospitality venues) for various reasons related to their perceived maliciousness. Big Tech was the primary stakeholder occasioning low trust in this regard. Participants tended to group Big Tech and private contractors as similar entities, and thus, some reported having generally low trust in both. Concerns were linked to the potential misuse of personal data and a perceived interest in getting involved with the T&T app only for profit. Similarly, when comparing small and large hospitality venues, the participants reported lower trust in the latter because of the potential for using T&T data for their own benefit:

It just doesn’t seem feasible to me that somebody such as a big company would be involved without getting something in return. There must be some sort of profit-making.

Participant B; decreased trust in Big Tech; change factor of 25

#### 4c: Small Venues, Local Councils, and the NHS

Conversely, the remaining stakeholders were considered not particularly malicious; however, their specific conditions such as bad outcomes, poor infrastructure, or decentralized procedures were sources of mistrust in them having the capability to adequately handle T&T activities. Participants mentioned trusting the NHS slightly less because of the rising COVID-19 cases. Some participants pointed out the poor treatment of personal data handled at small venues when manually checking in. In fact, most changes in trust to stakeholders occurred in regard to small venues (Figure 3). Similarly, participants recognized that local councils have less budget and poor communication with the centralized government:

I would say just from kind of experience that small businesses have a harder time doing data security and data protection work and that larger companies tend to have much bigger infrastructure for doing that.

Participant I; neutral trust in most stakeholders; change factor of 4

Despite participants’ concerns about how these stakeholders managed T&T and other pandemic-related matters, there was a general feeling of trust in them regarding their intentions. In fact, participants strongly empathized with small hospitality venues, and although they stated that their T&T procedures were not ideal, the harsh conditions they have been through with the pandemic were acknowledged, and thus, their efforts in following guidance were recognized. Similarly, participants considered that local councils made good efforts in handling pandemic issues at their local level and were perceived as more consistent than the central government. Some participants also expressed an existent or renewed general trust and appreciation for the NHS, as it had been put through difficult times:

The smaller they are the more sorry I feel for them. They kinda had to do their best. And I remember the time when we still had to sign manually which I assume must have been a nightmare. Especially considering they would have to close down if they didn’t have it. So I do trust them...I don’t think data was misused.

Participant B; increased trust in small venues, local councils, and NHS; change factor of 25

#### 4d: General Mistrust and Uncertainty Regarding Institutions Involved in T&T

Participants reported an overall mistrust of the “whole system,” on occasions specifically referring to the central government (as detailed in *T3: General Low Trust in the Government Owing to Poor Pandemic Handling, Including Procurement of the T&T App* section) and at other times conflating Big Tech organizations and other government dependencies. Further, other participants stated a general mistrust of real-world project development, pointing out gaps and malpractices, as well as general concerns regarding personal data treatment regardless of the institution. Nonetheless, these participants acknowledged that some compromises were needed for practical reasons for getting through the pandemic:

I wouldn’t say it’s about trusting the app. I just think the whole system’s a bit broken.

Participant F; decreased trust in small venues and the UK government; change factor of 18

### T5: Over Time, Public Consciousness and Compliance Have Lessened Regarding the Pandemic

#### 5a: Compliance Has Generally Drifted Away Over Time

This subtheme is substantial. Participants reported varying degrees of compliance with the use of the T&T app as well as other social distancing and isolation guidance. The degree of participants’ compliance with the guidance was based on internal and external factors. Participants reported following the pandemic measures, including the use of the T&T app to protect others, mentioning a sense of social responsibility. Although participants reported complying with the T&T app despite seeing it as flawed or untrustworthy, they still believed it should be used:

My loss of privacy in the bigger scheme of things is not as important for this period of time than potentially protecting the grandma next door. So, I downloaded [the app] despite my concerns.

Participant G; initial mistrust of data used responsibly and securely that remained unchanged, but high motivation of use to protect self and others; change factor of 10

On the other hand, when participants reported low compliance, they mentioned a combination of reasons, including the relaxation of the rules and the inconsistency and lack of coherence of guidance. People mentioned still relying on other physical measures such as testing and mask wearing, as well as a sense of security from being vaccinated. Participants also adapted the rules and guidance according to their own common sense; that is, what they viewed as the most effective or convenient solution for their specific situation:

It seems to be a combination of the vaccine, a combination of frustration over the length of the pandemic, or just that sheer, “I’ve gotta live my life.” And, for some friends and families, it’s financial pressures.

Participant E; initial negative trust in app that remained broadly unchanged; change factor of 9

I was every day in a class of 30 kids with no social distancing. It kind of became a bit of what’s the point?

Participant F; decreased perceived usefulness to self by 4 points and decreased trust that app is reliable and effective by 2 points; has deleted the app; change factor of 18

First time I had to use my Covid passport and stuff like that to get into places I was really happy, I just decided you know what, “I’m now in control of this myself, I’m not having the app.”

Participant D; decreased perceived usefulness to self and decreased trust in app effectiveness and trustworthiness; has deleted it; change factor of 9

I wanted to comply, to self-isolate. And I’d have to cancel the hotel, the trains, and it means a cost for me. So yeah, I did not comply. But we took the test, and we were tested negative, so we proceeded with our holidays.

Participant A; decreased trust that people will download the app; change factor of 12]

Interestingly, participants described experiences of compliance fading over time. These experiences referred to participants’ own compliance behavior changing, as well as other external factors such as the perceived behavior of the general public. Moreover, participants consistently reported observing venues not complying or prompting customers to follow T&T measures anymore, such as manually entering their details or checking in using the T&T app. They further expressed decreased visibility of QR codes at venues. Some participants believed that this was because T&T came across as not being needed anymore, and others said it reflected how it went out of public consciousness over time (see *5d: Media Exposure Has Decreased* section), although it could still be potentially useful:

The QR codes are still going on. I don’t think many people are using them. I do try to do them when I remember...and quite honestly, I find myself forgetting.

Participant C; increased trust that people will download the app and isolate; change factor of 8

It [T&T App] feels less important now it’s less visible. It’s significantly less visible, and I see many of my friends and family pretty much unaware of that test and trace is still going almost. The other things are because they know the visibility of the whole engine has gone, I still think it could be very important in tracking down outbreaks or in spotting new variants.

Participant E; decreased motivation of use to protect self; change factor of 9]

#### 5b: Early Interest and Curiosity in the T&T App Has Not Sustained

The participants also expressed dedicating some time to investigating on the web about the T&T app when it was first announced and released and were mainly curious about T&T app functionality. Some participants were concerned about data protection and thus closely followed media reports about T&T app development. However, participants also acknowledged that they stopped reading about the app, both because there were not as many media reports as at the beginning of the pandemic and because some eventually lowered their use of the T&T app:

Early doors I did, so when it first came out and the conversations were around, cause they partnered with like Google and a couple of other tech companies, and I was interested in the conversations about how much personal data was in there, how would be used, well like protections…So I was interested at that point, I can’t say I’ve read anything about the Covid-19 app in the last six months.

Participant H; mix of positive and negative responses that broadly remained unchanged; change factor of 4

#### 5c: Changed and Sustained Feelings Regarding T&T App

Finally, this subtheme was developed to reflect the participants’ changed and sustained feelings toward the T&T app. Participants who initially had negative expectations or assumptions about the app maintained those negative perceptions over time because of the government handling of the pandemic (described in *T3: General Low Trust in the Government Owing to Poor Pandemic Handling, Including Procurement of the T&T App* section) and the drop in T&T app use (described in *5a: Compliance Has Generally Drifted Away Over Time* section):

I was very sceptical and nothing has changed to make me less sceptical. I was very sceptical that it would work and you know, I’m, I consider myself very socially minded so I do believe in the good of society. I would care about the good of society, right? But I didn’t see how the test and trace app was actually for the good of society.

Participant I; initial negative perceptions of the T&T app that were maintained over time; never downloaded it; change factor of 4

Moreover, only one participant accounted for their positively changed trust in the T&T app over time, which was prompted by their questionnaire answers from a previous study:

I think a lot of it is because I took that survey really early on in getting the app and at the time I do think there were more problems with it. Bugging out or crashing or being a bit crap. And it’s kind of proven itself and had a couple of improvements overtime. So I’ve probably yeah, I’ve probably grown to think the app is better than I did when it first launched for it seemed a bit more slapdash.

Participant H; increased trust in app reliability; change factor of 4

#### 5d: Media Exposure Has Decreased

Although some participants reported media content adding to people’s negative perception of the T&T app, over the last year, the media content regarding T&T has decreased in favor of other topics considered more sensational. Some participants believed that the media and the government should increase exposure of the T&T app so that people are reminded of its existence and thus are prompted to use it:

I think that then it says on the UK Government to talk less about it. So it makes me, and maybe people, less concerned about it. So maybe if you just keep it in the same level. I mean, keep informed, persuade us to use the T&T app or to still doing the measures. They talk less about it, I think.

Participant A; decreased trust in small and large venues, positive unchanged trust in the UK government; change factor of 12

Didn’t think it was such a newsworthy thing anymore because there was, I mean pick, take your pick in all the things, in the scandals and what people do. And it is also not very juicy reporting to kinda “Oh we’re still using the app,” “keep using app”…There’s much more juicy [stuff] to read about so, I think it just became boring probably.

Participant G; initial negative trust in most stakeholders that remained unchanged; change factor of 10

## Discussion

### Principal Findings

A total of 5 themes were developed from the qualitative interview data, finding multiple reasons for changes in use of the T&T app. These are also reflected on the varied change factor in survey responses over time, the lowest being 4 points and the highest being 25 points. The largest contributors to change over time were the flaws experienced when using the T&T app (T1) and the lack of trust in the UK government because of how the COVID-19 pandemic was handled (T3). Other factors influencing trust included perceived usefulness and functionality of the app (T2), trust in stakeholders (T4), and public consciousness and compliance lessening over time (T5).

The results of this study elucidate on the concept of sustained use in the context of DCT, which for the T&T app consisted of a range of actions, some more passive than others; for instance, keeping Bluetooth tracing on or more actively scanning the QR codes at public venues. In this paper, we move beyond the TAM model [24] and instead adopt the concepts proposed by the TAL model (ie, the transition from preuse acceptance to initial and sustained use) [26] to explain participants’ experiences with the T&T app over time, which are influenced by their perceived usefulness and ease of use among other factors such as trust in the stakeholders involved. After a year of use, the NE of the T&T app wore off, as expressed in T5, in line with the literature [28]. Personal and social motivations to use the app have also changed (Figure 2), which are some of the factors that lead to sustained use of technology [29]; however, a few participants continued using it as it was a requirement for their job.

Trust is a complex topic but was generally reported to be influenced by a combination of perceived intentions and competence. Over this year, participants’ trust in involved stakeholders has also slightly changed according to Figure 3—trust in government (partly because of the scandals discussed in the introduction), large venues, and Big Tech decreased; trust in local councils and NHS increased; and trust in small venues increased for some participants and decreased for others (T3 and T4). There were some slight differences between the trust scores given in the surveys and the findings from the interviews. Furthermore, when the survey trust scores were averaged from both time points, most changes were not substantial (Figures 3 and 4), which further establishes the variable and subjective nature of trust. Trust in the stakeholders that form the app’s ecosystem influences trust in the app and its uptake [22,23]. The combination of the NE “wearing off,” lack of personal and social motivation for app uptake, and general low trust in T&T app stakeholders were reasons given by participants explaining their change in the use of the T&T app, as markedly evidenced by 2 of the participants deleting the app between the initial survey and the interview.

Studies investigating attitudes toward DCT apps have identified that people were positive about and intended to use them to help mitigate the spread of COVID-19 and protect others [3,11], which is broadly reflected by the statements provided by the participants of this study (Figure 3). Nonetheless, our results suggest that although social influences can be a motivator for adoption [7,10,16], changes in the use of the T&T app were occasioned by several factors such as experienced and perceived flaws, mistrust surrounding the whole app ecosystem, and everyday life practicalities and contingencies. Then, this study both confirms the intention-behavior gap identified in previous studies of DCT [11] and contributes to providing some of the reasons for its occurrence.

In line with the fifth theme developed in the thematic analysis, media representation and concern regarding the pandemic lessened over time, which appeared to have a direct effect on the behavior of participants. Although the initial intention to use the T&T app was positive as discussed earlier, the normalization of the pandemic in the media, along with a growing sense of pandemic fatigue, led to decreased use or deletion of the T&T app. Normalization and pandemic fatigue were therefore 2 key factors that had an impact on the compliance and behavior surrounding the use of the T&T app, despite the initial intention from participants to continue using the app. A further explanation for the lack of trust and poorly sustained use of the T&T app could be a “learned helplessness” developing in individuals because of consistent failures from both the UK government and from the technological capabilities of the T&T app. “Learned helplessness” is a learned state that develops from powerlessness arising from uncontrollable traumatic events, leading to the general belief that a situation is unchangeable [40]. As the COVID-19 pandemic was out of anyone’s individual control, and efforts to reduce the spread of the virus were appearing unsuccessful owing to the rise in cases, it is possible that people began to feel a sense of learned helplessness, which in turn led to complacency with using the T&T app. These explanations are consistent with previous findings, which highlight that a decrease in concern, low trust in political systems, and complacency can negatively affect the adoption of DCT apps [41,42].

Measures to stop the spread of COVID-19, like the uptake of DCT, are of a collective nature owing to the behavior of the virus. This tension between the need for a collective response and the individual-based design of the T&T app is shown in subtheme 1c. Fischer [43] demonstrated that the individual-collective nature of a society influences its collective actions regarding COVID-19 behavior, where more economically advantaged and individualistic societies have weaker collective action properties such as this study’s context (United Kingdom). Thus, the cultural context of the United Kingdom could be another factor influencing the intention-behavior gap identified in this study.

Finally, as some have started to point out [16], the results of this study divert from previous work reporting privacy and security concerns as major barriers to the adoption and use of DCT [3,4,44-47]. Although some participants stated having such worries, the perceived benefit of DCT overtook them. This occurrence may also be explained by the normalization of affective discomfort [48], by which people continue using apps despite considering them as dubious. Hence, although privacy and security may play an important role in the initial adoption and use, in the long term, these concerns moved to the background for our participants, possibly facilitated by a lack of major data breaches taking place. Furthermore, this study expands on the reasoning for mistrust in governments deploying DCT, beyond worries of massive surveillance [3,46]; as elaborated in T3, it is also constructed by people’s assessment of the government’s capabilities for managing the pandemic and creating and managing the T&T app.

### Practical Implications

Although this study operates in the specific context of the United Kingdom, several implications and lessons can be learned from the individual and collective experiences of people with the T&T app after a year of deployment. First, participants encountered by themselves, or as well-known social occurrences, a number of flaws with the app ranging from technical issues to little consideration of user diversity and how the app would be used in different situations. Moreover, the participants expressed a lack of clarity and certainty in their understanding of how the app works. Therefore, although people have good intentions to support society (Figure 3), or even if the apps are marketed to appeal to good citizenship and collectivism [7,10,49], our study suggests that if people do not see how DCT is achieving such a goal, sustained use becomes hindered. Alternatively, people who continue using the app do it despite not being sure if their actions are contributing to controlling the pandemic. Although this research suggests such implications, it is important to maintain that these conclusions were gathered from a small sample and cannot therefore be widely generalized.

The implication for future DCT systems is that besides considering a range of real-world scenarios (eg, multioccupant or shared-wall households) and a diverse set of users (eg, frontline workers), they must provide further contextual information to explain to users how decisions are being made by DCT apps and ensure transparency of the technical (eg, false positives) and practical (eg, effectiveness at large) matters. Moreover, this study shows that the deployment of DCT apps should go hand-in-hand with other measures to avoid provoking perceptions of uselessness. At a very minimum, DCT apps should keep being promoted over time by the organizations involved in deploying them. Other steps to improve sustained use could be taken by exploring the design of DCT that addresses the loss of NE and how trust in the whole ecosystem (app, organizations, and other users) can be strengthened.

Finally, participants’ accounts of their experiences using—or not using—the T&T app beg the question whether DCT is effective or needed at all [50]. This study aligns with findings from Tretiakov and Hunter [16], in which DCT actual use declined when the alert or risk levels were low. As we move into the endemic stage of COVID-19, the practical application of proximity-based DCT needs to be reassessed and must work in combination with other physical measures, such as vaccines and testing, that give people more reassurance and clearer results upon which they can act. Some directions include the development of hybrid contact tracing systems that integrate the participation of human contact tracers in the whole ecosystem [51].

### Limitations

There are some limitations of this research that should be highlighted. First, participants in the interview study were somewhat homogeneous demographically and could be considered a small sample size. This research only gathered qualitative data from 1 frontline worker (for as much as we know from the interview discussions, in which affected occupations were likely to be discussed), and all the participants had received higher education degrees (Table 1). Therefore, this sample and the subsequent thematic analysis may not be representative of a diverse population. In addition, Dowthwaite et al [19] identified statistically different responses for Black, Asian, and minority ethnic participants in their survey study. This could not be investigated in this study because only 1 out of 9 participants identified as member of the Black, Asian, and minority ethnic group; thus, these differences should be explored in future research.

Moreover, although participants were asked about the impact of the UK COVID-19 vaccination rollout on trust, the data on this were not substantial enough to form a robust theme. The impact of vaccinations was only mentioned by 1 participant in theme 5. This is not to say that vaccination rollout did not influence how people perceived the dangers of COVID-19 and the role of preventative measures, just that the individuals in this study did not focus on the topic when discussing their views on the T&T app and the factors influencing trust. It could be argued that many participants in this research were angered and frustrated by the UK government’s poor handling of the pandemic, which overpowered the positive influences such as the vaccination rollout in the United Kingdom.

Another limitation of this research is the lack of justification for the questionnaire scores at time point 1. As only quantitative data were gathered at the first time point, the authors could only truly compare the quantitative data longitudinally. The thematic analysis should only be considered longitudinal in a retrospective manner for descriptive explanations (participants were asked to reflect on how their trust and general views of the T&T app had changed over time). Consequently, this may have resulted in the possibility of recall bias and inaccurate portrayals of experience.

Finally, this study is geographically limited to the specific UK context, in which a centralized government and public health system exist. Thus, it could be argued that the findings of this research are only transferable to places with similar political, cultural, and health systems, if at all.

### Conclusions

To conclude, this research aimed to understand how the use of the T&T app and trust changed over time. By conducting interviews and exploring survey answers approximately 1 year apart, we found multiple reasons for changes in trust and diminishing use. For instance, the 2 largest contributors to change were the perceived flaws in the T&T app and a lack of trust in the UK government owing to the way the pandemic was handled. In addition, multiple factors impacted the participants’ compliance with the app. Initial NEs occurred with the T&T app, which lessened over time as a concern and media representation of the pandemic decreased and a new norm was established. These findings are an important initial step for future technology and app design and to increase understanding around how the general public perceives and trusts in the technology used for health care, and which factors influence the uptake and sustained use of DCT apps.

## Appendix

Multimedia Appendix 1 Statements presented in the web-based questionnaire and revisited in the interviews.

Multimedia Appendix 2 Participants’ details, attitudes, and changes from the initial questionnaire to interview.

Multimedia Appendix 3 List of themes and subthemes generated through thematic analysis.

## Abbreviations

DCT: digital contact tracing
NE: novelty effect
NHS: National Health Service
T&T: Test & Trace
TAL: technology acceptance life cycle
TAM: technology acceptance model
