# Supplementary material for: Understanding Trust and Changes in Use After a Year With the NHS COVID-19 Contact Tracing App in the United Kingdom: Longitudinal Mixed Methods Study
Source: J Med Internet Res. 2022 Oct 14;24(10):e40558. doi: 10.2196/40558 (PMC9578414; doi:10.2196/40558)
Supplement: Multimedia Appendix 1 [file jmir_v24i10e40558_app1.docx]

**Multimedia Appendix 1. Statements presented in the web-based questionnaire and revisited in the interviews.**

| **Question text** | **Response options** |
| --- | --- |
| **Experience of the pandemic:** |  |
| Thinking of the Coronavirus pandemic, which of the following, if any, apply to you?   - I have tested positive for COVID-19 - A member of my family has tested positive for COVID-19 - A close friend has tested positive for COVID-19 - A member of my household has tested positive for COVID-19 - I have been asked to self-isolate | Yes/No |
| **The COVID-19 App:** |  |
| Have you downloaded the NHS COVID-19 App on your smart phone | Yes, and I still have it on my phone  Yes, but I deleted it  No, but I intend to  No, and I don’t intend to |
| To what extent do the following describe why you downloaded the app?   - Because the government told me to - To help the NHS - To help protect myself - To help protect my friends and family - Because it will reduce the spread of the virus - To help protect broader society - Because it is a requirement of my job - Because everyone else is | 1 = Strongly disagree  2 = Somewhat disagree  3 = Neither agree or disagree  4 = Somewhat agree  5 = Strongly agree |
| If the participant has deleted the app  Which, if any, of the following reasons describe why you deleted the app   - It didn’t work on my phone - I didn’t know how to use it - I chose not to take part in contact tracing in this was - I didn’t think it would be effective - I don’t want to be tracked - I don’t want to be told to self-isolate - I don’t trust the people who built the app | Yes/No |
| Have you opened the app and had a look around? | Yes/no |
| Have you used the app for venue check-in with a QR code? | Yes/no |
| Have you made use of the ‘Check symptoms’ section of the app? | Yes/no |
| Have you got ‘Contact tracing’ switched on in the app | Don’t know/Never/Sometimes/Always |
| Has the app notified you to self-isolate?   - To what extent, if at all, did/would you follow this? | Yes/No   - Not at all/ Very little / Somewhat / Very much / Entirely |
| Which of the following is closest to your understanding of how decisions to send a notification to self-isolate are made via the app? | - Decisions are made by humans - Decisions are made by both humans and the app - Decisions are made by the app only |
| **Features of the App:** |  |
| To what extent do you agree or disagree with the following statements?   - I understand how the NHS COVID-19 app works - I am concerned about how my data will be used by the app - The app is useful to me personally - The app is useful to wider society - It is important to me that I can get an explanation for any information given to me by the app - It is important to me that I can verify the notifications from the app are authentic - The regulations governing the creation of the app are sufficient - It is important to me to be able to speak to a person about any advice given by the app - It is important to me that I can opt-in and opt-out of contact tracing | 1 = Strongly disagree  2 = Somewhat disagree  3 = Neither agree or disagree  4 = Somewhat agree  5 = Strongly agree |
| **Trust in the App:** |  |
| To what extent do you agree or disagree with the following statements?   - I trust that the data collected by the app is used responsibly - I trust that the data collected by the app is stored securely - I feel that the app is reliable - I trust that the app will do what it is supposed to do - I think the NHS COVID-19 app is basically trustworthy - I think that other people will download the app - I trust that other people will self-isolate if told to do so by the app - I trust that my data will be deleted when the app says it will - It is important to me that I trust the app in order to use it | 1 = Strongly disagree  2 = Somewhat disagree  3 = Neither agree or disagree  4 = Somewhat agree  5 = Strongly agree |
| **Trust in test and trace** |  |
| Please consider the following groups with regards to their involvement in the Test and Trace system. To what extent do you agree or disagree that you trust each group?   - The big tech companies, such as Google and Apple - Private contractors, such as Serco - Small hospitality venues, such as independent pubs and cafes - Large hospitality venues, such as chain restaurants - The UK Government - The Local Council - The NHS | 1 = Strongly disagree  2 = Somewhat disagree  3 = Neither agree or disagree  4 = Somewhat agree  5 = Strongly agree |
