# Supplementary material for: Understanding Trust and Changes in Use After a Year With the NHS COVID-19 Contact Tracing App in the United Kingdom: Longitudinal Mixed Methods Study
Source: J Med Internet Res. 2022 Oct 14;24(10):e40558. doi: 10.2196/40558 (PMC9578414; doi:10.2196/40558)
Supplement: Multimedia Appendix 2 [file jmir_v24i10e40558_app2.docx]

**Multimedia Appendix 2. Participants’ details, attitudes, and changes from the initial questionnaire to interview.**

| **Participant A**    32-years-old, Asian, male    Downloaded the app and still has it on his phone | A close friend had tested positive for Covid-19 prior to the first questionnaire with no additional experiences. He feels more strongly now that they downloaded the app to help the NHS (2 to 5); he also feels more strongly that it is important he can opt-in and out of contact tracing (3 to 5). His initial trust in the app was neutral, which overall hasn’t changed although he now has less trust that other people will download the app (4 to 2). His trust in small hospitality venues (4 to 1) and larger hospitality venues (4 to 2) has also decreased, but trust in other institutions remains neutral or moderate. |
| --- | --- |
| **Participant B**    31-years-old, white, female    Downloaded the app and still has it on her phone | She had been asked to self-isolate prior to the questionnaire and no additional points were raised since. She feels more strongly now that she downloaded the app because the government told her to (3 to 5), and less that it is to protect themselves (5 to 3), to help broader society (5 to 2), or because everyone else is (5 to 2). She also feels less strongly now that the app is useful to her personally (5 to 3), or that the regulations governing the app are sufficient (5 to 2). Her initial trust in the app was low to neutral, and this has remained the same although she now has less trust that other people will download the app (4 to 2) and feels less strongly that she needs to trust the app to use it (5 to 3). Her trust has increased in small hospitality venues (2 to 4), the local council (2 to 4), and the NHS (3 to 5), but lowered in the big tech companies (3 to 1). Trust in other institutions remains low. |
| **Participant C**    29-years-old, white, male    Downloaded the app and still has it on his phone | He has had no close experience of the pandemic, has had no change in opinion about why he has the app, and no real change in opinion about the technology surrounding the app. His initial trust in the app was neutral to moderate and he has had an overall increase in trust in the app, especially increasing in trust that most people will download the app (2 to 4) and that most people will self-isolate if told to do so (2 to 4). His trust in the NHS has increased a lot (1 to 5). His trust in the government and private contractors remains low, and moderate in the other institutions. |
| **Participant D**    43-years-old, white, female    Downloaded the app before completing the first questionnaire but has since deleted it | She has had no specific close experience of the pandemic, and feels less strongly now that the app was useful to them (4 to 1), or that it is important to be able to speak to a person about notifications (4 to 2). She had neutral trust initially in the app, and this has decreased slightly. She trusts less that the app does what it is supposed to (3 to 1) and that the app is basically trustworthy (3 to 1). Her trust in the UK government remains low, private contractors neutral, and other institutions moderate. |
| **Participant E**    49-years-old, white, male    Downloaded the app before completing the first questionnaire and still has it on his phone. | At the time of the first questionnaire a close friend had tested positive for Covid-19 and no additional points were raised. He feels less strongly now that he downloaded the app to protect himself (5 to 3), but more strongly that it is a requirement for his job (1 to 4). He is now less concerned about how his data will be used by the app (4 to 2). He had low to neutral trust in the app initially, and this has decreased, especially a lowering in trust that the data collected by the app is stored securely (4 to 2). His trust in big tech companies, private contractors, and the UK government remains low, the trust in other institutions remain neutral to moderate. |
| **Participant F**    43-years-old, white, female    Downloaded the app before completing the first questionnaire but has since deleted it | She has had no specific close experience of the pandemic. She downloaded the app but has have since deleted it. She now feels much less strongly that the app is useful to her (5 to 1) or wider society (5 to 3), or that the regulations surrounding the creation of the app are sufficient (5 to 2), but she feels that it is more important to her that they can opt-in and -out of contact tracing (4 to 2). She had moderate trust in the app initially and this has decreased, especially feeling less that the app is reliable (4 to 2), or that it does what it’s supposed to do (4 to 2). Her trust has also decreased in small hospitality venues (4 to 2) and the UK government (4 to 1). Her trust in other institutions remains moderate, and high in the NHS. |
| **Participant G**    30-years-old, non-binary    Downloaded the app before completing the first questionnaire and still has it on their phone. | They have had no specific close experience of the pandemic. They feel less strongly now that they downloaded the app to help the NHS (5 to 3) or because everyone else is (3 to 1) and more because it is a requirement for their job (1 to 3). They also feel more strongly now that they understand how the app works (2 to 4), and less that it is important to them to be able to speak to a person about notifications (5 to 3). They had low trust in the app initially, and their overall trust is the same, but they now feel that it is less important that they trust the app in order to use it (3 to 1). Their trust in small hospitality venues has decreased (4 to 2). Trust in other institutions remains low, and moderate in the NHS. |
| **Participant H**    36-years-old, non-binary    Downloaded the app before completing the first questionnaire and still has it on their phone. | At the time of the first questionnaire a close friend had tested positive for Covid-19 and no additional points were raised. They have had no real change in opinion about why they have the app, but they feel more strongly now that it is important that they can opt in and out of contact tracing (2 to 4). They had neutral trust in the app initially, and this has increased, especially feeling more that the app is reliable (2 to 4), that it does what it is supposed to do (2 to 4), and that most people will download it (3 to 5). Their trust in institutions remains low for most institutions and moderate for the UK government and NHS. |
| **Participant I**    49-years-old, white, female    Never downloaded the app and does not intend to | At the time of the first questionnaire a close friend had tested positive for Covid-19 and no additional points were raised. She had low initial trust in the app, and this has increased slightly, with an increase trust that most people will self-isolate (1 to 3) and that it is important to her to trust the app to use it (3 to 5). Her trust in institutions remains neutral. |
