# Supplementary material for: Understanding Trust and Changes in Use After a Year With the NHS COVID-19 Contact Tracing App in the United Kingdom: Longitudinal Mixed Methods Study
Source: J Med Internet Res. 2022 Oct 14;24(10):e40558. doi: 10.2196/40558 (PMC9578414; doi:10.2196/40558)
Supplement: Multimedia Appendix 3 [file jmir_v24i10e40558_app3.docx]

**Multimedia Appendix 3. List of themes and subthemes generated through thematic analysis.**

| **Theme** | **Subtheme** | **Abbreviated Subtheme (as used in the paper)** |
| --- | --- | --- |
| **Theme 1:** Flaws of the T&T App perceived and experienced by participants | Subtheme **1a:** T&T App inefficiency is a combination of lack of technical advance and lack of general public uptake | Subtheme **1a:** Lack of technical advance and lack of general public uptake |
|  | Subtheme **1b:** Lack of consideration of User Diversity | No change |
|  | Subtheme **1c:** T&T App designed for individuals rather than a collective effort | No change |
|  | Subtheme **1d:** Lack of clarity and certainty in understanding of the T&T App | No change |
|  | Subtheme **1e:** Suggestions given to improve the T&T App | No change |
| **Theme 2:** Trust in the T&T App differs based on perceived usefulness and functionality | Subtheme **2a:** Different perceptions about T&T App functionality and effectiveness translate into varying degrees of trust | Subtheme **2a:** Varying degrees of trust |
|  | Subtheme **2b:** Mistrust in T&T App due to flawed development and lack of explainability and transparency | Subtheme **2b:** Flawed development and lack of explainability and transparency |
|  | Subtheme **2c:** Varying preferences and trust in Human T&T and physical measures, although human guidance in T&T App is perceived as important | Subtheme **2c:** Human T&T, physical measures, and human guidance |
| **Theme 3:** General low trust in the government owing to poor pandemic handling, including procurement of the T&T App | Subtheme **3a:** Inconsistent and poor decision-making and lack of compliance from government leaders has caused low trust | Subtheme **3a:** Inconsistent decision-making and lack of compliance |
|  | Subtheme **3b:** Mistrust in government due to T&T App creation and data management | Subtheme **3b:** T&T app creation and data management |
|  | Subtheme **3c:** Tensions exist between enforcing the T&T App and opting out of tracing | Subtheme **3c:** Tensions between enforcing the T&T app and opting out of tracing |
|  | Subtheme **3d:** Participants reported general disapproval of the government’s actions during the pandemic | Subtheme **3d:** General disapproval of the Government’s actions during the pandemic |
| **Theme 4:** Varying degrees of trust in stakeholders | Subtheme **4a:** Participants’ trust in stakeholders can be seen as a relationship between perceived intentions and competence | Subtheme **4a:** Relationship between perceived intentions and competence |
|  | Subtheme **4b**: Big Tech, private contractors and large hospitality venues are considered more capable of handling pandemic issues, but participants mistrust their intentions | Subtheme **4b:** Big Tech, private contractors, and large hospitality venues |
|  | Subtheme **4c**: Small venues, local councils and the NHS are considered well-intentioned, but there is some mistrust in their ability to manage pandemic matters | Subtheme **4c:** Small venues, local councils, and the NHS |
|  | Subtheme **4d**: Some general mistrust or uncertainty regarding institutions involved in T&T, although compromises and shared responsibilities are acknowledged | Subtheme **4d:** General mistrust and uncertainty regarding institutions involved in T&T |
| **Theme 5**: Over time, public consciousness and compliance have lessened regarding the pandemic | Subtheme **5a:** Despite varying degrees of compliance being reported, compliance has generally drifted away over time | Subtheme **5a:** Compliance has generally drifted away over time |
|  | Subtheme **5b:** Participants reported early interest and curiosity in learning about T&T App, which has not been sustained over time | Subtheme **5b:** Early interest and curiosity in the T&T app has not sustained |
|  | Subtheme **5c:** Changes and sustained feelings regarding T&T App | No change |
|  | Subtheme **5d:** Media exposure over the last year regarding T&T has decreased, which affected people’s concern | Subtheme **5d:** Media exposure has decreased |
| **Theme 6:** Experiences with Isolation and Various Negative Emotions Evoked by the Pandemic | Subtheme **6a:** All participants isolated for varied reasons, despite no positive cases reported | - |
|  | Subtheme **6b:** Fear of getting pinged and infected, stress, loneliness and concerns of shortcomings and lack of understanding regarding vaccination | - |
